# Crohn’s Disease: From Immune Dysregulation to Intestinal Damage

**DOI:** 10.1007/s10753-026-02561-z

**Published:** 2026-07-11

**Authors:** Silvia Salvatori, Rachele Frascatani, Pasquale De Vico, Irene Marafini, Giovanni Monteleone

**Affiliations:** 1 Gastroenterology Unit, Fondazione Policlinico “Tor Vergata”, Rome, Italy; 2 Department of Systems Medicine, University of Rome “Tor Vergata”, Rome, 00133 Italy; 3 Department of Anaesthesia, University of Rome “Tor Vergata“, Rome, 00133 Italy

**Keywords:** Crohn’s disease, IBD pathogenesis, Immune dysregulation, Mucosal inflammation, Cytokines

## Abstract

Crohn’s disease (CD) is a long-lasting, recurring inflammatory bowel disease (IBD) marked by segmental, transmural inflammation that may affect any part of the gastrointestinal tract. Growing evidence suggests that CD does not originate from a single pathogenic mechanism, but instead results from the convergence of various biological disturbances that collectively disrupt intestinal homeostasis. Genetic alterations that weaken microbial detection, autophagy, and epithelial protection appear to establish initial vulnerabilities, enabling microbes to trigger exaggerated immune responses. This vulnerability is further exacerbated by environmental factors that intensify host microbial imbalance. The ensuing microenvironment fosters a significant production of effector molecules, which exacerbate the existing inflammation and activate stromal cells, which in turn drive tissue remodeling and structural complications such as ulceration, strictures, and fistulas. In this article, we synthesize the knowledge on the molecular and cellular mechanisms involved in CD pathogenesis, emphasizing the dynamic interplay between immune and stromal components that contributes to a disease-permissive environment. We also examine key inflammatory pathways and signaling networks involved in disease progression and discuss how these mechanistic insights inform existing and emerging therapeutic strategies.

## Introduction

Crohn’s disease (CD), a principal form of inflammatory bowel disease (IBD) in humans, is characterized by chronic, relapsing, tissue-destructive inflammation of the gastrointestinal tract. Unlike ulcerative colitis (UC), which is limited to the rectum and colon and primarily affects the mucosal layer, CD can involve any tract of the gastrointestinal tract, with segmental inflammation affecting all layers of the intestinal wall. This deep inflammatory process results in aphthous and longitudinal ulcers, fibrostenotic strictures, and penetrating lesions (i.e., fistulas and abscesses), which can lead to clinical consequences (e.g., malabsorption, malnutrition, bowel obstruction, and an increased risk of intestinal cancer) [1].

A central hallmark of CD pathogenesis is the disruption of intestinal homeostasis, a tightly regulated state that normally ensures harmonious interactions between the host immune system, the intestinal epithelium, and the commensal microbiota. In healthy individuals, epithelial cells form a dynamic physical and biochemical barrier, while innate and adaptive immune responses maintain tolerance towards resident microbes and mount rapid, controlled responses against pathogens [2, 3]. In CD, defects in epithelial barrier integrity facilitate microbial translocation across the mucosa, thus triggering exaggerated immune responses that culminate in excessive production of inflammatory cytokines and chemokines [4].

Growing evidence suggests that the development of CD cannot be attributed to a single pathogenic defect. Instead, it results from the complex interplay of genetic susceptibility, environmental exposures, and host-microbial interactions, which establishes a disease-permissive microenvironment that drives persistent inflammation, progressive tissue remodeling, and the development of the characteristic complications of CD [1, 4].

This article provides an updated overview of the molecular and cellular mechanisms underlying CD pathogenesis, with particular emphasis on the key inflammatory pathways and signaling networks that orchestrate immune activation, stromal cell engagement, and tissue remodeling. Understanding these interconnected processes is essential for elucidating disease progression and guiding the development of targeted therapeutic strategies to restore intestinal homeostasis and prevent long-term complications.

## Genetic and Environmental Priming of Immune Dysregulation

More than 240 susceptibility loci have been identified in IBD through genome-wide association studies (GWAS). Some are common to both IBD types, while others are unique to either CD or UC [5]. Approximately one-third of CD patients carry single-nucleotide polymorphisms (SNPs), within or near the leucine-rich repeat domain, of NOD2. In particular, SNP8, SNP12, and SNP13, which are associated with CD in Caucasians but not Asians, result in amino acid changes (Arg702Trp, Gly908Arg) or a frameshift (1007insC) leading to a truncated protein. NOD2 variants are linked to ileal CD and, consequently, their presence identifies individuals at increased risk of fibrostrictures [6, 7].

NOD2 controls the production of α-defensin by Paneth and immune cells [8], and NOD2 SNPs are linked to decreased NF-kB activation and defensin synthesis in CD patients [9]. Nod2-null mice do not spontaneously develop pathology. However, after infection, NOD2-deficient mice develop ileitis, which is marked by enlarged Peyer’s patches and elevated levels of T helper type 1 (Th1) cytokines. The inflammatory phenotype in these mice is reversed when NOD2 function is restored [10]. These data are in line with the demonstration that NOD2 controls the myeloid cell response to bacteria and the production of interleukin-12 (IL-12) [11, 12], the master inducer of Th1 cell differentiation [13].

NOD2 mediates the protective effects of Enterococcus faecium in the gut. Specifically, bacterial DL-endopeptidase SagA-driven NOD2 activation in myeloid cells induces IL-1β secretion, thereby increasing the proportion of IL-22-producing CD4 + T helper (Th) cells and innate lymphoid cells that promote tissue repair. Interestingly, the protective effects of Enterococcus faecium are abolished in mice carrying the IBD-associated NOD2 gene variant [14].

NOD2 is also essential for autophagy, a process necessary for eliminating bacteria and maintaining cellular health. It recruits the autophagy protein ATG16L1 to sites of bacterial invasion. Variants in the ATG16L1 gene, particularly the rs2241880 mutation, are associated with CD. These gene alterations impair autophagy, resulting in the accumulation of harmful substances [15–17]. Furthermore, defective autophagy in individuals with CD leads to a failure to effectively eliminate microbial pathogens, exacerbating inflammation and increasing the likelihood of bacterial persistence, particularly adherent-invasive Escherichia coli (AIEC), a pathogen closely associated with CD [18]. ATG16L1 also interacts with murine norovirus and helps prevent necroptosis in Paneth cells by maintaining mitochondrial function [19, 20]. Another CD susceptibility locus is Immunity-Related GTPase M (IRGM), which regulates autophagy and inflammasome signaling. Deficiency or dysregulation of IRGM results in the accumulation of damaged mitochondria and reactive oxygen species, which can potentiate NLRP3 inflammasome activation, increased secretion of IL-1β and IL-18 and enhanced pyroptosis. Furthermore, impaired IRGM-mediated bacterial clearance may contribute to persistent microbial stimulation of the mucosal immune system, thereby exacerbating disease progression and chronic intestinal injury in CD [21]. GWA studies have identified a connection between the TNFSF15 gene, which encodes tumor necrosis factor-like ligand 1 A (TL1A), and increased susceptibility to both CD and UC [22]. Interestingly, in Korean patients with CD, non-risk allele homozygotes of TNFSF15 SNPs rs6478108 and rs4574921 were identified as independent genetic predictors for the development of strictures/non-perianal penetrating complications and perianal fistulas, respectively [23].

For a detailed description of the genetic alterations associated with CD, the reader is directed towards recent reviews [24–26].

Early life factors, such as being born by caesarean section, antibiotic use, and formula feeding, contribute to the development of an abnormal microbiome, which can impede the establishment of immune tolerance and increase the risk of CD later in life [25, 27, 28]. For example, caesarean section births disrupt the natural colonization of microbiota, and early antibiotic administration can reduce microbial diversity and encourage the proliferation of pathobionts [29, 30].

Additional environmental factors contributing to an increased risk of CD include dietary habits in western cultures, such as high salt intake [31], high saturated fat [32], high refined sugar consumption [33], and food preservatives [34–36], which all alter gut microbiota by providing an advantage for bacteria that degrade mucus, are resistant to bile acids, and therefore increase the permeability of the epithelial barrier [37]. Furthermore, pesticides can alter the composition and function of the gut microbiota, increase intestinal permeability, and promote oxidative stress, potentially increasing the risk of CD onset and progression [38]. Cigarette smoking is another important environmental risk factor for CD. It increases oxidative stress and damages the repair process of the epithelial cells. Furthermore, cigarette smoking alters the composition of the gut microbiota in humans and animals, allowing for the proliferation of harmful bacteria [39]. Cigarette smoking worsens Paneth cell defects in CD patients and mice that possess mutations in the ATG16L1 gene [39]. Patients with CD who carry the ATG16L1 mutation and smoke have a higher risk of postoperative recurrence compared to those who either do not smoke but have the ATG16L1 mutation, or who smoke but do not carry the mutation [39].

Taken together, these observations suggest that multiple genetic alterations and environmental factors contribute to triggering an abnormal immune response in the gut of patients with CD.

## Innate Immune Dysregulation

The body’s primary defense against microbial threats is the innate immune system. In CD, the function of innate immune cells is both impaired and exaggerated, resulting in a prolonged inflammatory response. Macrophages, which normally respond to pathogens by ingesting and destroying them, exhibit deficiencies in recognizing and clearing microorganisms in CD. Simultaneously, immunostimulatory molecules derived from the gut microbiota bind to toll-like receptors and activate signaling pathways (e.g., NF-κB, MAP kinases) in various antigen-presenting cells (APCs), including CD14 + myeloid cells recruited from the systemic circulation and dendritic cells (DCs). This activation leads to increased production of effector cytokines (e.g., IL-6, IL-34, TNF, and TL1A), which amplify mucosal inflammation and contribute to the disruption of epithelial integrity [40–43]. These cells also produce master cytokines, such as IL-12 and IL-23, which influence Th-cell polarization [44], as well as IL-1β and IL-18, as a result of inflammasome activation. This activation results in auto-processing of caspase-1 and subsequent maturation of IL-1β and IL-18 [45] as well as cleavage of gasdermin D that induces pores in the cell membrane thereby promoting pyroptosis [46]. Research using experimental colitis models has demonstrated that inhibiting inflammasome components, such as NLRP3 or caspase-1, reduces gut inflammation [47, 48]. In contrast, mice lacking GBP5, a positive regulator of inflammasome activation, show resistance to colitis [49]. Consistently, administration of anti-mature IL-18 antibodies or IL-1β blockers ameliorates experimental colitis in mice [50, 51], as well as treatment of children with refractory very early onset IBD with IL-1β blockers appears to be safe and effective [52].

Increasing evidence indicates that, regardless of the driving stimulus, several factors amplify the innate immune response in CD. For example, OTUD5, an enzyme that stabilizes proteins by cleaving ubiquitin linkages, is overexpressed in the inflamed gut of CD patients and enhances TNF production [53]. Excessive cytokine production in CD also depends on reduced SIRT1 expression, a protein deacetylase that inhibits immune pathways. Consistently, activating SIRT1 with Cay10591 reduces NF-κB activation and alleviates colitis in mice [54]. Additionally, defective expression of programmed death ligand 1 (PD-L1) on APCs impairs T regulatory cell (Treg) function, leading to a breakdown of immune tolerance [55]. In CD, there is over-expression of Smad7, an inhibitor of TGF-β signaling. Knockdown of Smad7 in CD mucosal cells with a specific antisense oligonucleotide (ASO) reduces inflammatory cytokine production, and administration of the Smad7 ASO in mice with CD-like experimental colitis attenuates ongoing inflammation [56]. Consistently, selective deletion of Smad7 gene in DC enhances TGF-β1 signaling and promotes Treg differentiation, thus protecting mice against intestinal inflammation [57].

CD-associated inflammation is also marked by a reduced expression of other immune counter-regulators, such as thymic stromal lymphopoietin (TSLP), IL-10, and IL-25. TSLP, produced primarily by intestinal epithelial cells, promotes a tolerogenic phenotype in DCs, limiting the production of IL-12 and IL-23 [58, 59]. IL-10, secreted by Tregs, regulatory B cells, macrophages, and ILCs, acts directly on intestinal macrophages and DCs to suppress the production of inflammatory cytokines (e.g., TNF, IL-1β, IL-6, and IL-23) [60]. IL-25, which is produced by epithelial cells, tuft cells, and other stromal populations, restrains inflammatory responses by inhibiting the activation of ILC1s and ILC3s and myeloid cells [61–63].

The aryl hydrocarbon receptor (AHR), a ligand-activated transcription factor expressed by multiple innate immune populations including macrophages, DCs, and ILC3s, is also downregulated in CD [52]. AHR signaling normally suppresses NF-κB-driven inflammatory pathways, limits the production of TNF and IL-6 by myeloid cells, and supports the maintenance of IL-22-producing ILC3s that contribute to epithelial barrier integrity and antimicrobial defense [64]. In addition, AHR promotes the differentiation and stability of Tregs while restraining excessive Th17 responses [65, 66].

Overall, these findings contribute to delineating a scenario in which a complex network of signals enhances the innate immune response in CD, leading to an excessive production of inflammatory cytokines.

## The Role of Neutrophils and Innate Lymphoid Cells in Tissue-Damaging Inflammation in Crohn’s Disease

Accumulating evidence suggests that excessive neutrophil recruitment and activation in response to microbial antigens in the lamina propria contribute significantly to mucosal damage in CD. The active phases of CD are characterized by the accumulation of neutrophils within the mucosal layer, with their presence correlating with disease severity. In contrast, the resolution of neutrophil infiltration is associated with improved clinical outcomes, such as reduced corticosteroid use and fewer hospitalizations [67–69]. Calprotectin, a calcium- and zinc-binding protein predominantly produced by neutrophils, is often elevated in faecal samples of IBD patients and serves as a useful biomarker for diagnosis and monitoring disease progression [70]. The accumulation of neutrophils in the affected areas of CD patients relies on various mechanisms. The recruitment of neutrophils from the bloodstream is favored by signaling molecules largely released by epithelial cells [e.g. IL-8, IL-6, IL-33, CXCL5, CXCL7, CXCL10, CCL20, leukotriene B4, hepoxilin A3, and matrix metalloproteinases (MMPs)] [71]. When arrived in the inflamed tissue of CD patients, neutrophils tend to survive longer due to defective apoptosis, a phenomenon influenced by cytokines, hypoxia, extracellular acidosis, and bacterial products (e.g., LPS, lipoproteins, peptidoglycans, and lipoteichoic acids) [72, 73]. The resolution of mucosal inflammation involves specialized pro-resolving mediators (SPMs), a class of lipids synthesized from omega-3 and omega-6 polyunsaturated fatty acids. SPMs interact with specific G protein-coupled receptors (GPCRs) to halt neutrophil migration and induce their apoptosis [74, 75]. The expression of chemokine-like receptor 1, also known as chemerin receptor 23 (ChemR23), a GPCR targeted by resolvin E1 and highly expressed in macrophages, DCs, and neutrophils [76, 77], is up-regulated in the inflamed mucosa of CD patients unresponsive to biologic treatments and associated with increased neutrophil accumulation [78]. Consistently, studies in colitic mice showed that treatment with an anti-ChemR23 antibody facilitated the transition of pro-inflammatory macrophages to an anti-inflammatory state, promoting neutrophil apoptosis and clearance [78].

Within the gut mucosa, neutrophils secrete various proteolytic enzymes and reactive oxygen species, which can damage the epithelial barrier [79]. Neutrophils also contribute to CD pathogenesis by secreting cytokines and chemokines (e.g., CXCL1, CXCL2, CXCL5, IL-8, IL-1β, and IL-17), which recruit other immune cells such as monocytes, T cells, and NK cells [80]. Neutrophils can respond to microbial stimuli and other factors (e.g. cytokines) secreted within the inflamed gut by forming neutrophil extracellular traps (NETs), an extracellular network composed of DNA, histones, and antimicrobial proteins that trap and neutralize pathogens [81]. A key step in the process of NET formation, or NETosis, is the citrullination of histones H1, H3, and H4 by peptidyl arginine deiminase 4 (PAD4), which facilitates chromatin decondensation and expulsion [82]. The triggering receptor expressed on myeloid cell-1 (TREM-1) is another component of NETs, and pharmacologic inhibition or genetic ablation of TREM-1 reduces NETosis thus limiting NET-dependent pathology [83]. NET formation is enhanced in the mucosa of IBD patients, including those with CD, and correlates with disease activity [84]. Experimental evidence suggests links NETs to gut pathology, as NETs can trigger epithelial cell apoptosis, compromising the intestinal barrier and worsening inflammation in the CD-like TNBS-induced colitis model [84].

ILCs are essential components of the immune system, particularly in mucosal immunity. They provide a rapid immune response, complement adaptive immune cells, and are critical for maintaining intestinal homeostasis [85, 86]. The gut-associated lymphoid tissue (GALT) contains various subsets of ILCs, primarily ILC1s, ILC2s, and ILC3s, which respond to signals from the microbiota, cytokines, and environmental cues [87]. In patients with CD, a distinct population of intraepithelial ILC1s characterized by IFN-γ production, expression of CD127, lack of NKp44, and low c-kit levels is selectively expanded following stimulation with IL-12 and IL-18 [88]. This subset is absent from the intestines of alymphoid mice reconstituted with a human immune system but emerges in the colons of mice subjected to dextran sulfate sodium (DSS)-induced colitis [88]. Although these cells are classified as ILC1s, their low-level expression of RORγt suggests that they may originate from RORγt-positive ILC3s under inflammatory conditions. A distinct ILC1-related population, referred to as intraepithelial ILC1-like cells, expresses NKp44, NKp46, CD56, CD103, granzyme, and perforin, and resides within the intestinal epithelial layer. Unlike conventional ILC1s, these cells lack IL-7Rα expression and do not depend on IL-15 for their development or survival. Nevertheless, they remain functionally responsive to IL-12 and IL-15, resulting in enhanced IFN-γ secretion [89]. Intraepithelial ILC1-like cells are enriched in the inflamed intestinal tissue of CD patients and have been shown to drive pathology in anti-CD40-induced colitis in Rag1-deficient mice [89].

ILC3s are primarily localized to the intestinal mucosa, where they secrete multiple effector molecules, including RegIIIβ, RegIIIγ, IL-17 A, IL-22, and GM-CSF. Their protective activity is largely attributed to IL-22. This pathway has been demonstrated in mice colonized with segmented filamentous bacteria or invasive Escherichia coli, as well as in animals treated with acetate or propionate, ligands of free fatty acid receptor 2, conditions under which ILC3-derived IL-22 supports epithelial repair [90–92]. The beneficial effects of IL-22 on epithelial cells require activation of AHR [93]. Consistently, AHR-deficient mice display increased susceptibility to colitis and Citrobacter rodentium infection due to reduced intestinal IL-22 production [64, 94, 95]. Colonization with Lactobacillus reuteri D8 further highlights this pathway, as bacterial production of indole-3-aldehyde, an AHR agonist, enhances IL-22 secretion by ILC3s and alleviates DSS-induced colitis [96].

Mechanistic studies indicate that optimal cytokine production by ILC3s relies on distinct intracellular signaling pathways. Although RORγt deficiency does not impair IL-22 production required for maintaining intestinal homeostasis, it significantly reduces the secretion of heparin-binding epidermal growth factor by activated ILC3s, a factor necessary for protection against DSS-induced colitis [97]. In addition, post-transcriptional regulation through the p38α–eIF6–Nsun2 signaling axis is essential for the appropriate expression of protective cytokines, and disruption of this pathway increases susceptibility to intestinal inflammation [98]. SIRT6 has been identified as a negative regulator of IL-22 production in ILC3s; selective deletion of SIRT6 enhances IL-22 secretion without altering ILC3 numbers, thereby conferring increased resistance to DSS-induced colitis [99].

Recent evidence has identified a population of RORγt-expressing ILCs in anti-CD40-induced colitis that co-express markers associated with both ILC2s and ILC3s and produce IL-10. Targeted deletion of IL-10 in these cells exacerbates both innate and adaptive immune-driven colitis, highlighting their regulatory potential [100].

The abundance of IL-17-producing ILC3s in the intestine is positively regulated by neuropilin-1 (NRP1), a transmembrane protein whose expression is elevated in CD. Loss of NRP1 reduces both the frequency of intestinal ILC3s and their capacity to produce IL-17 A, leading to alterations in the gut microbiota and attenuation of DSS-induced colitis [101]. Furthermore, IL-23 and IL-1β activate the IRE1α/XBP1 stress-response pathway in ILC3s through mitochondrial reactive oxygen species, resulting in enhanced production of IL-17 A and IL-22. IRE1α/XBP1-expressing ILC3s are enriched in experimental colitis and in inflamed intestinal tissue from CD patients, where their frequency correlates positively with responsiveness to ustekinumab, an antibody targeting IL-12/IL-23p40 [102]. Collectively, these findings indicate that ILC3s adopt distinct functional programs in response to environmental and inflammatory cues, thereby playing context-dependent roles in both the maintenance of intestinal homeostasis and the development of gut pathology.

## Adaptive Immune Dysregulation in Crohn’s Disease

The inflamed mucosa in CD is heavily infiltrated by adaptive immune cells, including T and B lymphocytes, memory T cells, T follicular helper cells, and stem-like CD4⁺ T cells. These cells are mainly recruited from the circulation in response to chemokines produced in the inflamed tissue. T cell trafficking to the gut also depends on interactions between integrins (e.g., α4β7) and endothelial ligands [i.e., Mucosal Vascular Addressin Cell Adhesion Molecule 1 (MAdCAM-1), intercellular adhesion molecule-1 (ICAM-1) and vascular cell adhesion molecule-1 (VCAM-1)]. Lymphocyte accumulation is further enhanced by apoptosis resistance, which is mainly mediated by locally produced cytokines (e.g., IL-6, IL-15, and IL-21) [103, 104]. Mucosal T cells display activation markers, express various transcription factors, and secrete multiple effector cytokines. Historically, CD was considered a Th1-mediated disease, with high IFN-γ production and elevated Th1 transcription factors (i.e., Stat4, T-bet) [105, 106]. Recent studies, however, revealed that CD mucosa harbors additional Th subsets, such as Th9 cells, Th17 cells and T follicular helper cells, which likely amplify inflammation [107, 108].

In CD, the major driver of Th1 differentiation is IL-12 [44], whereas Th17 cell differentiation and expansion require TGF-β1 and Stat3-activating cytokines (i.e., IL-6, IL-21, and IL-23) [108, 109]. Microbiota and microbial metabolites, including short-chain fatty acids, secondary bile acids, and indole derivatives, further shape Th17 development. Indeed, germ-free mice display reduced intestinal T cells, restored by colonization with commensals. Transferring IBD-associated microbiota into germ-free mice increases Th17 cells, decreases Tregs, and exacerbates colitis [110]. Specific microbes, such as segmented filamentous bacteria (SFB), and bacterial components, such as SFB flagellins, strongly induce Th17 responses via epithelial production of serum amyloid A (SAA), which can substitute for TGF-β1 in Th17 induction [111, 112]. Other bacteria, including Propionibacterium, Prevotella, and Lactobacillus casei, similarly promote Th17 accumulation [113–115]. CD mucosa contains also high numbers of IL-17-producing CD8 + T cells (Tc17 cells), which express a distinct immune signature (CD6high, CD39, CD69, PD-1, CD27low) linked to disease activity [116]. Microbiota-reactive CD4⁺ memory T cells are normally present in the gut but expand in CD, where they produce high levels of IFN-γ, IL-13, IL-17 A, and TNF-α, and their abundance correlates with disease activity. CD8⁺ memory T cells, predominantly in the epithelium, express markers like CD49a, CD69, and CD103, which facilitate tissue retention. Cytokines such as TGF-β1, TNF-α, IL-6, and IL-33 promote memory T cell differentiation, whereas IL-12 and IFN-β inhibit it [117].

A disruption of the balance between effector T cells and Tregs also marks CD-associated inflammation. Treg frequency is often increased, yet effector T cells may resist to Treg-mediated suppression due to Smad7-mediated inhibition of TGF-β1 signaling [56]. Tregs can also acquire effector-like properties (e.g., Th17-like cells), thus reducing their suppressive capacity [118]. Finally, in CD, Tregs express lower α4β7 integrin, limiting gut homing and function [119].

B cells and plasma cells accumulate in the CD mucosa, and are supposed to contribute to CD-associated tissue damage. In the inflamed ileum and colon of CD patients, IgA production is reduced, whereas IgG production is increased compared with that in normal controls [120]. IgG4 + plasma cells are abundant in areas with high histologic score of fibrosis [121], although it remains to be determined whether these cells contribute to fibrogenesis or merely represent an epiphenomenon of the fibrotic process. CD patients produce elevated levels of IgG and IgA antibodies against microbial antigens, including Saccharomyces cerevisiae, flagellin, and E. coli [122–124]. Granzyme B–expressing plasmablasts, which can kill intestinal epithelial cells in vitro, infiltrate the mucosa of CD patients [107]; however, the contribution of these cells to tissue damage in CD remains to be validated.

Ectopic lymphoid formations, also known as tertiary lymphoid tissues, are observed in the inflamed intestines of CD patients. These formations, usually not found in healthy tissues, serve as locations where B cells and T cells become activated and interact, thereby enhancing the immune reaction. The development of these clusters is believed to be influenced by cytokines (e.g., TNF-α and IL-17) [125], with their occurrence linked to disease severity and connected to resistance against specific treatments [126]. Additionally, these formations facilitate the attraction of B cells and their maturation, into IgG4 plasmablasts. IgG4 plasma cells produce platelet-derived growth factor (PDGF), which could stimulate PDGF-receptor-expressing fibroblasts and myofibroblasts, thereby resulting in uncontrolled fibrosis [127].

## Interactions Between Immune Cells and Stromal Cells are Crucial in Expanding the Mucosal Inflammation and Promoting Tissue Damage

**Fig. 1:**
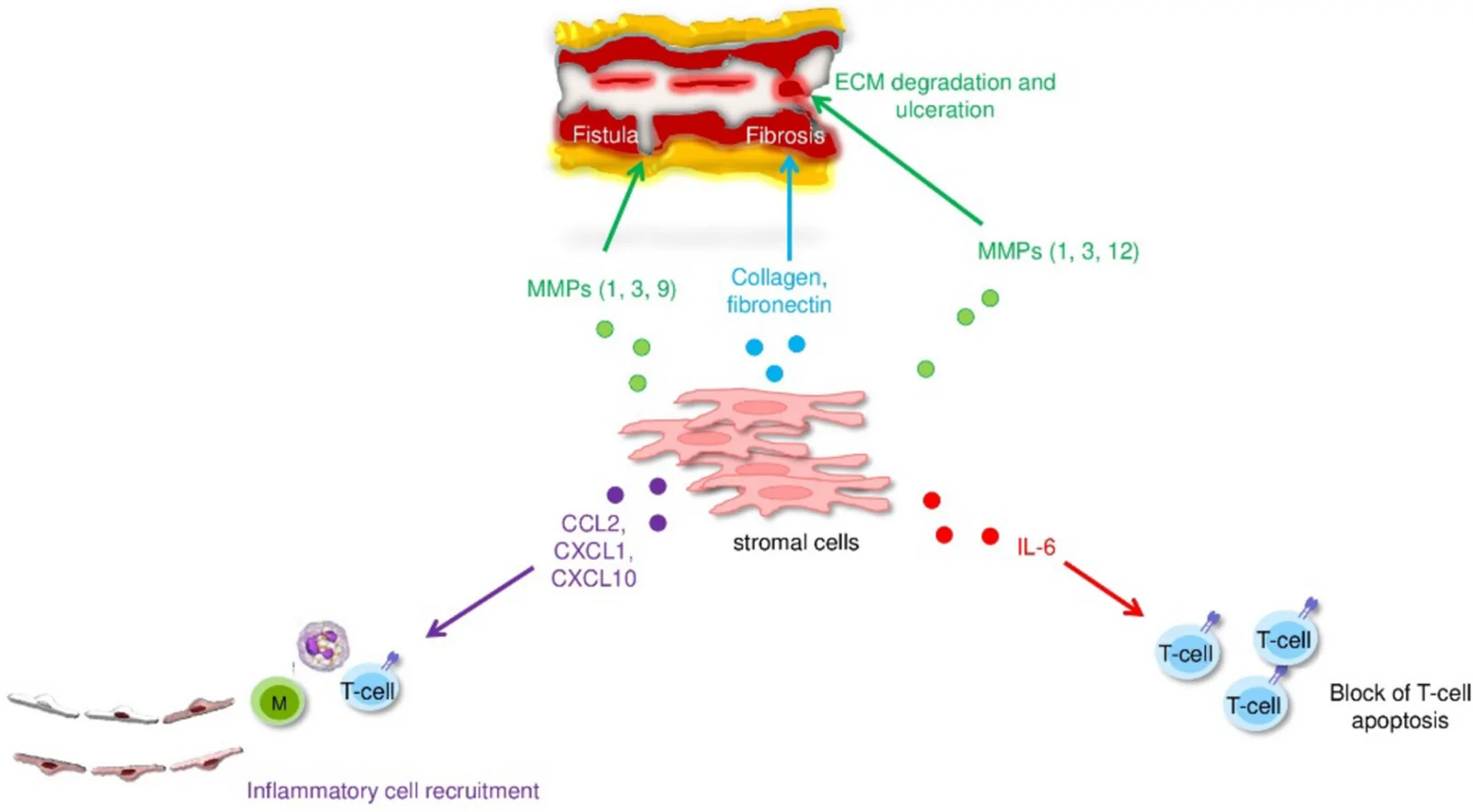
Schematic representation of the role of stromal cells in Crohn’s disease (CD). In the CD mucosa, stromal cells amplify inflammation by releasing cytokines, such as interleukin-6 (IL-6), which inhibit T-cell apoptosis, as well as chemokines—including chemokine (C-C motif) ligand 2 (CCL2), C-X-C motif chemokine ligand 1 (CXCL1), and CXCL10—that promote the recruitment of immune cells from the circulation to sites of inflammation. In addition, stromal cells are a major source of matrix metalloproteinases (MMPs), enzymes responsible for extracellular matrix degradation, thereby contributing to ulcer and fistula formation. Furthermore, stromal cells secrete profibrogenic factors, such as collagen and fibronectin, which facilitate the development of intestinal strictures

The stromal section, consisting of fibroblasts, myofibroblasts, endothelial cells and smooth muscle cells offers both integrity and functional assistance to the intestinal tissue. Fibroblasts and myofibroblasts react strongly to cues and play a role, in tissue repair and healing of wounds. In CD, upon activation by pro-inflammatory cytokines (e.g., TNF-α, IL-1β, IL-17, and IL-21), fibroblasts proliferate, migrate, and differentiate into myofibroblasts, which play a role in the breakdown of the epithelial barrier and the development of ulcers in CD. This process is mainly driven by their secretion of MMPs, such, as MMP-1, MMP-3 and MMP-12, which degrade the extracellular matrix (ECM) thus inducing the detachment of epithelial cells (Fig. 1).

As noted above, the release of these MMPs is primarily induced by cytokines produced by immune cells, underscoring how interactions between immune and stromal cells can exacerbate tissue injury [128–131]. Mucosal regions in CD showing epithelial damage are enriched in IL-11-positive inflammatory fibroblasts, suggesting that these cells may contribute to ulcer formation [132, 133]. These cells also expressed cytokines and chemokines induced in proximity to T cells or epithelial cells, suggesting that inflammatory fibroblasts help orchestrate immune responses [133]. Indeed, intestinal fibroblasts release chemokines like CCL2, CXCL1 and CXCL10 that draw cells, including monocytes, T lymphocytes, and neutrophils to inflammatory regions [134, 135]. Furthermore, fibroblasts secrete cytokines such as IL-6, which support the survival and maintenance of inflammatory cells, within the impacted tissue [136] (Fig. 1).

Activated fibroblasts produce also ECM components such as collagen, fibronectin, and laminin [137], thus contributing to the formation of strictures. Creeping fat, the expansion and invasion of mesenteric adipose tissue in and around regions of the inflamed gut, is a feature of CD. Notably, creeping fat contains a population of fibroblasts expressing CTHRC1 that exhibit a transcriptional profile enriched for YAP/TAZ pathway components, cytoskeletal remodeling genes, and profibrotic effector programs [138]. These cells can respond to mechanosensitive priming with enhanced deposition of ECM thus exacerbating tissue rigidity and fibrostricturing processes [138].

Single-cell and spatial transcriptomic analyses of CD samples revealed that intestinal strictures are marked by a depletion of intraepithelial CD8⁺ T cells, absorptive enterocytes, and resident fibroblasts, alongside an expansion of immune cell populations. These include IgG⁺ plasma cells, CCR7-hi CD4 + T cells consistent with naïve or central memory phenotypes, and IL-11–expressing inflammatory fibroblasts. Analysis of ligand-receptor interactions suggests that IL-1 drives inflammatory fibroblast polarization, while CCL2 facilitates monocyte co-localization [133]. Accordingly, serum IL-11 levels are higher in stricturing than in non-stricturing CD and correlate with an increased risk of surgical intervention [139].

Mucosal myofibroblasts expressing MMP-1, MMP-3, and MMP-9 are also abundant in fistulous tracts, supporting their involvement in the development of such lesions. Recent single-cell RNA sequencing (scRNA-seq) studies of rectal tissue from perianal fistulas have shown active interaction between myeloid and stromal cells and highlight CHI3L1 as one of the top upregulated genes in stromal cells within fistulas. These cells express destructive and fibrotic gene signatures, implying they have dual functions in tissue remodeling and disease processes [140]. Further scRNA-seq studies by McGregor et al. has identified nine distinct fibroblast subsets within fistulating CD tissue. Notably, a subset of fibroblasts, termed FAS cells, displays gene expression profiles associated with fibrosis, tissue remodeling, and the secretion of multiple cytokines and chemokines, suggesting their involvement in ECM remodeling, tissue repair, and immune modulation. Analysis of transcription factors showed that TWIST1 activates fibrotic mechanisms in these cells while the transcription factor Odd-skipped related 2 (OSR2) controls pathways that encourage ECM breakdown aiding epithelial recovery and tissue remodeling. FAS cells share also characteristics with inflammatory fibroblasts, including CHI3L1 expression, and can establish localized inflammatory niches through interactions with SPP1-positive macrophages and other immune populations [141].

More recently, Gudiño and colleagues demonstrated that the rectal mucosa of patients with perianal fistulising CD displays distinct cellular and transcriptomic changes driven by TL1A signaling in CD4⁺ T cells. In this context, TL1A promotes lymphotoxin-β production which, together with IL-22, induces a TNF-α–independent gene program associated with perianal fistulising disease, characterized by the upregulation of MMPs in fibroblasts [142].

Collectively, these studies indicate that inflamed CD tissue harbors heterogeneous fibroblast populations, with some subsets contributing to epithelial injury and others participating in tissue repair, fibrosis, and fistula tract formation. Therapeutically, selectively targeting pathogenic fibroblast subsets may offer a promising approach for the treatment of distinct CD phenotypes.

## Novel Therapeutic Targets

The growing resolution with which the mucosal immune system can be analyzed (e.g., single-cell transcriptomics, spatial profiling, and high-parameter cytometry) has reshaped our understanding of CD and is driving innovation in therapeutic strategies. Historically, treatment has focused on targeting inflammatory cytokines or lymphocyte trafficking. Anti-TNF agents (e.g., infliximab, adalimumab, certolizumab pegol) remain widely used and highly effective for inducing and maintaining remission. The development of anti-IL-12/23p40 therapy (ustekinumab) and, more recently, selective IL-23p19 inhibitors (risankizumab, guselkumab, mirikizumab) reflects the centrality of the IL-23 axis in sustaining pathogenic Th17 and ILC3 responses. These agents have shown clinically meaningful rates of endoscopic and histologic improvement, supporting the relevance of this pathway across CD phenotypes. Anti-integrin therapy, particularly vedolizumab (α4β7 inhibitor), provides gut-selective immunosuppression by inhibiting T lymphocyte trafficking to the intestinal mucosa. Moreover, vedolizumab modulates innate immunity by altering macrophage populations and the expression of molecules involved in microbial sensing and chemoattraction [143]. By tracking intravenously administered fluorescently labeled vedolizumab in the gut, Gabriëls and colleagues showed that the drug binds to several immune cell populations, including plasma cells, macrophages, and eosinophils, whereas little or no binding to T cells was detected. These observations suggest that the therapeutic effects of vedolizumab are not mediated exclusively through T cells [144]. Additional benefit comes from small molecules, such as JAK inhibitors (e.g., upadacitinib) that suppress a wide cytokine network, while S1P receptor modulators (e.g., ozanimod) that restrict egress of lymphocytes from secondary lymphoid organs represent another valid therapeutic option [145]. Nonetheless, primary non-response and loss of response affect a substantial proportion of patients, thus indicating the necessity for novel treatments.

**Fig. 2:**
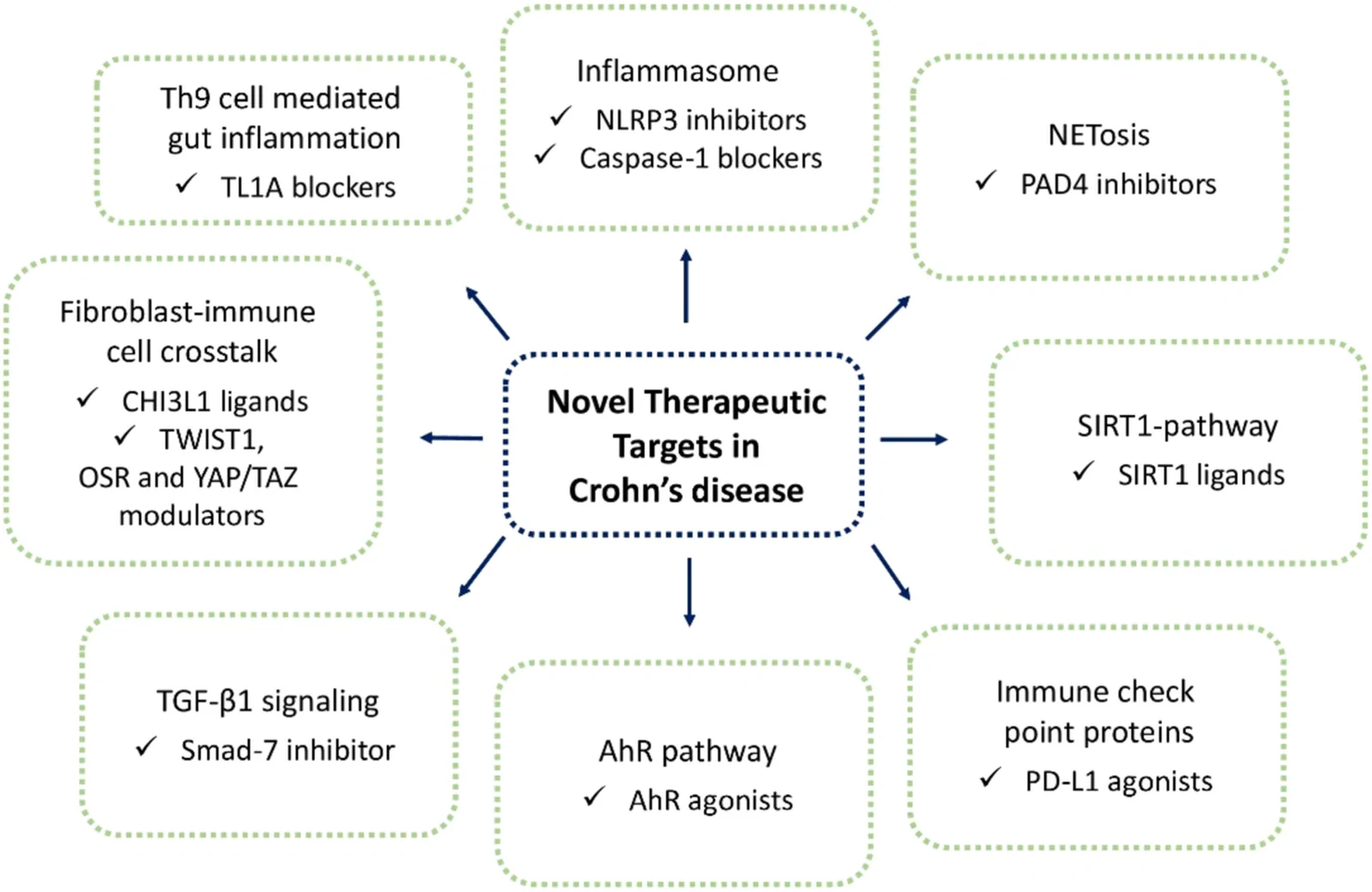
Emerging therapeutic targets for Crohn’s disease. AhR: Aryl Hydrocarbon Receptor, CHI3L1: Chitinase 3-Like 1, NLRP3: NACHT, LRR and PYRIN domain containing 3, PAD4: Peptidylarginine deiminase 4, PD-L1: Programmed Death-Ligand 1, SIRT1: Sirtuin 1, Smad-7: Mothers against decapentaplegic homolog 7, TGFβ1: Transforming Growth Factor β11, TL1A: Tumor necrosis factor-like ligand 1 A, TAZ: transcriptional coactivator with PDZ-binding motif, TWIST1: Twist Family bHLH Transcription Factor 1, YAP: Yes-associated protein

The main findings discussed in this article suggest that targeting additional effector molecules involved in CD pathogenesis could offer new therapeutic strategies, as illustrated in Fig. 2.

These approaches aim to intervene earlier in the inflammatory cascade or selectively reprogram cellular niches that sustain chronic inflammation. Several upstream innate immune nodes could be promising druggable checkpoints. For instance, NLRP3 inflammasome inhibitors, caspase-1 blockers, and PAD4 inhibitors targeting NETosis could help inhibit the initial cytokine bursts that activate downstream adaptive pathways and promote tissue destruction [47, 48, 146]. Further approaches include the targeting of intracellular kinases amplifying cytokine production, SIRT1-modulating compounds that restore metabolic restraint of innate activation, and agents that enhance PD-L1 expression or other tolerogenic signals on APCs [147–149]. The AhR pathway is another promising target, because its activation supports Treg differentiation, enhances epithelial barrier repair, and limits pro-inflammatory cytokine production [150]. Furthermore, approaches that normalize Th17/Treg balance, through the use of low-doses of recombinant IL-2 [151] are being evaluated for their capacity to restore physiological immune tolerance without global immunosuppression.

Restoring TGF-β1 signaling is another potential goal of CD therapies. Early phase 1 and phase 2 clinical trials have demonstrated both clinical and endoscopic efficacy of Mongersen, an oral Smad7 inhibitor, in patients with active CD [152–156]. However, a phase 3 trial was halted early after a futility analysis indicated that Mongersen was ineffective in these patients [157]. The reasons for the discrepancies across the clinical trials are not well understood, although recent evidence indicates that different batches of Mongersen used in the phase 3 study had chemical variations, with some failing to effectively downregulate Smad7 expression [158]. Therefore, new clinical studies using more potent Smad7 inhibitors are needed to further assess its role in CD.

One of the most significant conceptual advances is the recognition that stromal cell subsets, rather than being passive bystanders, actively orchestrate chronic inflammation, thus contributing to the formation of ulcers, fibrosis, and fistulas. This could inspire new therapeutic avenues aimed at disrupting fibroblast-immune cell crosstalk. Promising targets include CHI3L1 signaling, TWIST1-driven profibrotic programs, and fibroblast-derived chemokine networks (e.g., CCL2, CXCL10) that sustain immune infiltration [159–161]. The discovery of inflammatory fibroblast subsets, including FAS cells enriched in fistulizing disease, offers an opportunity to selectively inhibit or reprogram stromal populations responsible for maintaining pathogenic microenvironments. Similarly, approaches aimed at blocking SPP1⁺ macrophage-fibroblast interactions or modulating OSR2 or YAP/TAZ-regulated pathways may allow specific treatment of fibrosis and fistulas. Fibroblast activation protein, IL-7 receptor, and transcription factor AP-2 gamma could be additional targets to treat the fibrostricturing phenotype [162]. In this context, it is also important to note that growing evidence suggests that TL1A plays a role in propagating signals, which promote Th9 cell-mediated gut inflammation, fibrosis, and fistula formation [163]. Therefore, inhibiting TL1A may offer a promising strategy for managing inflammatory, fibrostricturing, and fistulizing CD [164, 165].

## Conclusions

Accumulating evidence reinforces the notion that CD does not arise from a single pathogenic mechanism, but rather, from the interplay of multiple biological pathways that collectively alter the intestinal homeostasis. Several genetic changes and environmental factors can promote defects in epithelial barrier integrity and microbial detection/response, leading to inappropriately controlled crosstalk between immune cells and non-immune cells, thus causing varying degrees of mucosal injury, fibrosis, and complications. Nonetheless, we do not yet know the exact sequence of molecular events that activate the pathogenic immune response, and whether the pathogenic immune alterations occur stably in all patients. Notably, the profile of effector cytokines can vary over time even in the same patient [166], and emerging evidence suggests that different immune responses can drive similar morphological lesions [167], thus explaining why not all patients respond to the current drugs.

The mechanistic insights emerging from recent studies emphasize the role of specific cell subsets in the propagation of gut-damaging immune responses, and suggest a shift in therapeutic strategies. For example, therapies like CD19 CAR T-cell therapy, which is showing promise in refractory UC, could represent a more targeted approach by selectively modulating immune responses that are resistant to current drugs [167, 168]. Additionally, restoring microbial-immune tolerance through microbiota-based interventions or antigen-specific therapies could offer another avenue for combating the pathological inflammation. An alternative strategy involves combination therapies that target multiple inflammatory pathways or simultaneously suppress effector signaling while restoring epithelial regulatory functions. At the same time, it is clear that a more comprehensive understanding of the complex biological mechanisms underlying CD is essential for the development of personalized and more effective therapeutic approaches tailored to the distinct pathophysiological characteristics of individual patients [169, 170].

## Data Availability

No datasets were generated or analysed during the current study.
